# Supplementary material for: Functional Division of Insect Blood Cells by Single-Cell RNA-Sequencing and Cell-Type-Specific FISH Markers
Source: Cells. 2025 Nov 22;14(23):1842. doi: 10.3390/cells14231842 (PMC12691282; doi:10.3390/cells14231842)
Supplement: Supplementary file 1 [file cells-14-01842-s001.zip › cells-4000769-supplementary.pdf]

Supplementary Information

Table S1. FISH probe sequences

Table S2. Uniquely expressed genes in 24 different hemocyte clusters

Table S3. Top three genes highly expressed in each hemocyte cluster of naïve *S. exigua* larvae

Table S4. Top three genes highly expressed in each hemocyte cluster of immune-challenged *S. exigua* larvae

Figure S1. Differential gene expression analysis in naïve and immune-challenged hemocytes. (A) Venn diagrams showing the unique expressed genes in naïve and immune-challenged larvae across hemocyte clusters. The numbers within each circle indicate the cluster-specific gene counts. (B) Bar plots showing the number of differentially expressed genes (DEGs) in each cluster, separated into upregulated (red) and downregulated (blue) genes upon immune challenge.

Figure S2. Heatmap of marker gene expression across hemocyte types. Heatmaps show the expression patterns of selected marker genes in different hemocyte populations of (A) naïve and (B) immune-challenged *S. exigua* larvae, including granulocyte (‘GR’), plasmatocyte (‘PL’), oenocytoid (‘OE’), and spherulocyte (‘SP’).

Figure S3. Unique probe signal across hemocyte types. (A) Hemocytes from L5 larvae were analyzed by FISH using probes for granulocytes (‘GR’), plasmatocytes (‘PL’), oenocytoids

(‘OE’), and spherulocytes (‘SP’). Each experiment included three biological replicates, and all hemocytes were scored as GR, PL, OE, SP, or “No signal.” Cells showing hybridization with two or more probes were annotated accordingly. The number of hemocytes analyzed varied among three replicates (‘Rep 1-Rep 3’). (B) Bar graph showing the percentage of unique FISH probe signals detected in the four hemocyte types. Data are presented as mean ± SE. Different letters above bars indicate statistically significant differences among hemocyte types.

Table S1. FISH probe sequences

FISH targets (Gene-hemocyte type)	Direction	Sequence (5'-3')
CecB1-GR	Antisense	FAM-TTTTCCCGAGTGTTTCTGTTCGT
	Sense	FAM-AAAACGAACAGAAACACTCGGGA
PMH-PL	Antisense	FAM-TTTTTGGCGACCTTTACCAGCAT
	Sense	FAM-AAAATGCTGGTAAAGGTCGCCAA
PPO2-OE	Antisense	FAM-TTTCACGGTCGGAGAAGTCCAAA
	Sense	FAM-AAATTTGGACTTCTCCGACCGTG
REPAT9-SP	Antisense	FAM-TTTGCAAACCTTCACCGAGTCGT
	Sense	FAM-AAAACGACTCGGTGAAGGTTTGC
PMH-PL	Antisense	Rhodamine-6G-TTTTTGGCGACCTTTACCAGCAT
	Sense	Rhodamine-6G-AAAATGCTGGTAAAGGTCGCCAA
PPO2-OE	Antisense	Cyanine3-TTTCACGGTCGGAGAAGTCCAAA
	Sense	Cyanine3-AAATTTGGACTTCTCCGACCGTG
REPAT9-SP	Antisense	Marina Blue-TTTGCAAACCTTCACCGAGTCGT
	Sense	Marina Blue-AAAACGACTCGGTGAAGGTTTGC
2-Oxo-nHC1	Antisense	FAM-TTTATCTGCTCAGGCGTCAGTTC
	Sense	FAM-AAAGAACTGACGCCTGAGCAGAT
DS-nHC2	Antisense	FAM-TTTAGTGCTATCGACTGACCGGC
	Sense	FAM-AAAGCCGGTCAGTCGATAGCACT
CP1-nHC3	Antisense	FAM-TTTTGCCACCTTCAGAGCGTTAG
	Sense	FAM-AAACTAACGCTCTGAAGGTGGCA
GPCR No9-nHC4	Antisense	FAM-TTTCTGGTCGTTCAGGCGGTAAT
	Sense	FAM-AAAATTACCGCCTGAACGACCAG
GPCR A3-nHC5	Antisense	FAM-TTTGTTCGACTTCTGCCCTTGGA
	Sense	FAM-AAATCCAAGGGCAGAAGTCGAAC

Table S2. Uniquely expressed genes in 24 different hemocyte types

Cluster (HC type)	Total genes	Unique genes	Genes
0 (OE1)	12,059	5	Cell division control protein 45 homolog, sex-lethal homolog, insulin-degrading enzyme, nuclear pore complex protein Nup85, zinc finger CCCH domain-containing protein 13-like
1 (nHC1)	9,423	1	2-oxo-4-hydroxy-4-carboxy-5-ureidoimidazoline decarboxylase-like
2 (GR1)	5,879	0	-
3 (GR2)	11,036	1	antennal esterase CXE14
4 (PL1)	10,204	42	transmembrane and TPR repeat-containing protein 1-like, U17490 cytochrome P450 (CYP4g15), homeobox protein 10-like, antennal esterase CXE10, UDP-glycosyltransferase 42C2, transmembrane protease serine 9-like, MAGUK p55 subfamily member 5, synaptic vesicle glycoprotein 2B-like, cytochrome P450 6B1-like, fork head domain transcription factor, ATP synthase subunit d, mitochondrial-like, putative inorganic phosphate cotransporte, myosin-11-like, mitochondrial cardiolipin hydrolase, FAS-associated factor 2, delta and Notch-like epidermal growth factor-related receptor, alkaline ceramidase, intraflagellar transport protein 74 homolog, cuticle protein 3-like, citron rho-interacting kinase, ankyrin repeat domain-containing protein 54, sperm flagellar protein 1-like, nuclear receptor subfamily 2 group C member 2-like, cytochrome p450 CYP6AB12 (CYP6AB12), suppressor of fused homolog, synaptic vesicular amine transporter, modular serine protease-like, transcription termination factor 5, beta-galactoside alpha-2,6-sialyltransferase 2, host cell factor-lik, arginine-hydroxylase NDUFAF5, scavenger receptor class B member 1-like, cadherin-23, probable RNA-binding protein 46, endocuticle structural glycoprotein SgAbd-5-like, histidine-rich glycoprotein-like, cuticle protein 19-like, histidine-rich glycoprotein-like, granzyme-like protein 1, UDP-glycosyltransferase 46A7
5 (PL2)	10,204	13	trypsin beta-like, SexiOBP13, agrin, probable 28S ribosomal protein S25, coatomer subunit delta, retinol dehydrogenase 13-like, signaling mucin HKR1-like, pupal cuticle protein 20-like, protein cortex-like, HIRA homolog, xanthine dehydrogenase, ubiquitin-like-conjugating enzyme ATG10, chaoptin-like

6 (nHC2)	3,811	46	neuropeptides capa receptor-like, beta-1,4-glucuronyltransferase 1, protein phosphatase 1 regulatory subunit 36, synaptic vesicle glycoprotein 2C-like, cAMP-dependent protein kinase catalytic subunit alpha-like, apyrase-like, NGFI-A-binding protein, general odorant-binding protein 19a-like, transmembrane protease serine 9-like, serine/threonine-protein phosphatase 6 regulatory ankyrin repeat subunit A, soluble scavenger receptor cysteine-rich domain-containing protein, orcokinin 1, enoyl-CoA delta isomerase 1, cuticle protein 16.5, sperm-associated antigen 6, trypsin-1, SWI/SNF-related matrix-associated actin-dependent regulator of chromatin, adipokinetic hormone 1, serine/threonine-protein kinase greatwall, Down syndrome cell adhesion molecule-like protein Dscam2, transcription factor HNF-4 homolog, RNA-binding protein fusilli, prolow-density lipoprotein receptor-related protein 1, methyl-CpG-binding domain protein 4-like, odorant receptor OR3, UDP-glycosyltransferase 33J3, zinc finger protein 600, leucine-rich repeat serine/threonine-protein kinase 1-like, serine protease snake-like, myb/SANT-like DNA-binding domain-containing protein 4, MFS-type transporter SLC18B1-like, retinol dehydrogenase 11-like, fatty acyl-CoA reductase wat-like, proton-coupled folate transporter-like, histidine-rich glycoprotein-like, septum formation protein Maf-like, platelet-activating factor acetylhydrolase IB subunit beta homolog, transcription termination factor, beta-1,3-galactosyltransferase brn-like, myelin transcription factor 1-like protein, DDB1- and CUL4-associated factor 7, dynein heavy chain 2, aldehyde dehydrogenase X, histidine-rich glycoprotein-like, equilibrative nucleoside transporter 1
7 (GR3)	3,902	1	inositol polyphosphate 1-phosphatase-
8 (GR4)	10,747	0	-
9 (nHC3)	9,227	0	-
10 (GR5)	10,418	3	glycine N-methyltransferase, zinc finger protein 706-like, glutathione S-transferase epsilon 1
11 (OE2)	9,728	1	protein Spindly
12 (GR6)	9,433	0	-
13 (OE3)	9,489	1	ester hydrolase C11orf54 homolog
14 (GR7)	9,503	7	transmembrane protein 135, guanine nucleotide-binding protein subunit beta-like protein 1, adenylyl cyclase 78C, cytoplasmic 60S subunit biogenesis factor, zinc finger protein 235, sulfotransferase 1 family member D1-like, alkylglycerol monooxygenase-like
15 (GR8)	8,065	2	odorant binding protein 18, serine protease snake-like
16 (GR9)	9,802	3	50S ribosomal protein L1, MDS1 and EVI1 complex locus protein EVI1-A, rRNA methyltransferase 3
17 (GR10)	7,536	8	S-adenosylmethionine sensor upstream of mTORC1, G patch domain and ankyrin repeat-containing protein 1, chemosensory protein CSP1, diapausin A6, protein KTI12, aldo-keto reductase AKR2E4-like, TATA-box-binding protein-like, transmembrane protein 181
18 (nHC4)	7,157	1	G-protein coupled receptor No9

19 (OE4)	11,557	234	apoptosis-stimulating of p53 protein, histone acetyltransferase KAT2A, paired box protein Pax-6, glutaredoxin domain-containing cysteine-rich protein, leucine-rich repeat-containing protein, transmembrane and TPR repeat-containing protein 1-like, proton-coupled amino acid transporter-like protein pathetic, elongation of very long chain fatty acids protein 4-like, gastrin/cholecystokinin type B receptor, gastrin/cholecystokinin type B receptor-like, mucin-5AC-like, nose resistant to fluoxetine protein 6, synaptotagmin-5, lipase member H-B-like, gamma-aminobutyric acid receptor subunit beta-like, branched-chain-amino-acid aminotransferase, proton channel OtopLc, delta-sarcoglycan, semaphorin-2A-like, LIM domain only protein 3-like, zinc transporter 9, teneurin-a, brain tumor protein, homeobox protein CDX-1, nephrin, BTB/POZ domain-containing protein 17, putative transporter svop-1, cadherin-like protein, lachesin, PAS domain-containing protein cky-1, natriuretic peptide receptor 2-like, UDP-glycosyltransferase 40F4, adenoma-associated protein homolog, PHD finger protein rhinoceros, ammonium transporter Rh type B, ribonuclease 3 , UDP-glycosyltransferase 43A2, semaphorin-2A, organic solute transporter alpha-like protein, fasciclin-2-like, zinc finger protein 383, cilia- and flagella-associated protein 61-like, mitochondrial ribonuclease P catalytic subunit, transmembrane protein 151B , UDP-glucuronosyltransferase 2C1, xanthine dehydrogenase 1-like, endothelin-converting enzyme 2-like, SKI family transcriptional corepressor 2, lachesin, giant-lens, disintegrin and metalloproteinase with thrombospondin motifs, adipokinetic hormone/corazonin-related peptide receptor variant I, ATP-dependent RNA helicase DHX34 , neurexin-1, hemicentin-2, kelch-like protein 13, putative epidermal cell surface receptor, dynein beta chain, synaptogenesis protein syg-2, flavin-dependent monooxygenase 1, transient receptor potential channel pyrexia, C-1-tetrahydrofolate synthase, WD repeat-containing protein 37, cell adhesion molecule Dscam2, 39S ribosomal protein L37, zinc finger protein 541, BTB/POZ domain-containing protein, insecticyanin 1 (Ins1), sodium/calcium exchanger 1, putative inorganic phosphate cotransporter, NFX1-type zinc finger-containing protein 1-like, cuticle protein 10.9-like, signal recognition particle receptor subunit beta, allatostatin C1 , serine--pyruvate aminotransferase, acyl-CoA Delta-9 desaturase, dual oxidase maturation factor 1, septin-2, multidrug resistance protein 1B-like, mucin-17, ATP-binding cassette subfamily C member4, potassium voltage-gated channel protein Shab, WW domain-binding protein 11 , BAI1-associated protein 3 , testis-expressed protein 47-like, collagen alpha-1(XVIII) chain, blue opsin, lipid droplet-regulating VLDL assembly factor AUP1, fumarylacetoacetase, kinesin-like protein unc-104, nuclear pore membrane glycoprotein 210, MOXD1 homolog 1-like, epidermal retinol dehydrogenase 2, neurofilament medium polypeptide, reversion-inducing cysteine-rich protein, zonadhesin-like, solute carrier organic anion transporter family member 4A1, arrestin domain-containing protein 4, solute carrier organic anion transporter family member 74D, peroxisomal acyl-coenzyme A oxidase 1, scarlet-like , tyrosine-protein kinase Dnt, tyrosine-protein kinase Drl, lachesin-like, cilia- and flagella-associated protein 57, cytosolic carboxypeptidase 4-like, cytochrome p450 CYP4M15, leucine-rich repeat-containing protein let-4, dynein heavy chain 8, axonemal-like, dynein axonemal heavy chain 8, protein rolling stone-like, leucine-rich PPR motif-containing protein, oxysterol-binding protein-related protein 1, Down syndrome cell adhesion molecule-like protein Dscam2, guanine nucleotide exchange factor subunit Rich, dynein axonemal heavy chain 10, thrombospondin type-1 domain-containing protein 4-like, proton-coupled folate transporter, sex peptide receptor-like, testis-specific serine/threonine-protein kinase 3-like, atrial

			natriuretic peptide receptor 1, synaptic vesicle glycoprotein 2C-like, orexin receptor type 1-like, enoyl-CoA hydratase, nucleoredoxin-like, alpha-tocopherol transfer protein-like, zinc finger protein Xfin-like, peripheral-type benzodiazepine receptor-associated protein, neo-calmodulin, putative chemosensory receptor 2, fatty acid synthase-like, glucose dehydrogenase, trichoplein keratin filament-binding protein, glucose dehydrogenase, fatty acid synthase-like, gustatory receptor 9 (GR9), bicaudal D-related protein, alpha-tocopherol transfer protein, wolframin, neuropeptide F receptor, cGMP-specific 3',5'-cyclic phosphodiesterase-like, cell adhesion molecule Dscam2, lachesin-like, homeobox protein orthopedia-like, potassium voltage-gated channel protein Shal, hemicentin-2, homeotic protein ocelliless, solute carrier family 2, mitochondrial DNA helicase, RNA polymerase II transcription subunit 14, methylcrotonoyl-CoA carboxylase subunit alpha, cyclic nucleotide-gated cation channel alpha-3, diuretic hormone 45, zinc finger protein DZIP1L, multidrug resistance-associated protein 4-like, serine/threonine-protein kinase, gamma-aminobutyric acid type B receptor, nose resistant to fluoxetine protein 6-like, PDZ domain-containing protein 8, voltage-dependent calcium channel type A, obstructor-E, tenascin-like, probable pathogenesis-related protein , ecdysone-induced protein , netrin receptor, tubulin polyglutamylase TTLL2, glutamine synthetase 2 cytoplasmic-like, pancreatic triacylglycerol lipase-like, mucin-5AC, ras-related protein Rab-3, RCC1 and FYVE domains-containing protein 1-like, dynein axonemal heavy chain, G-protein coupled receptor 158, metallophosphoesterase 1-like, hydrocephalus-inducing protein-like, MPN domain-containing protein, cysteine-rich venom protein, homeotic protein spalt-major, adenylate cyclase type 8, neuroligin-1-like, glial cells missing 2-like, tyrosine-protein kinase transmembrane receptor Ror-like, sodium- and chloride-dependent glycine transporter 1, putative leucine-rich repeat-containing protein, coiled-coil and C2 domain-containing protein 1-like, reticulocalbin-2, solute carrier family 23 member 2, sulfotransferase 1E1, endothelin-converting enzyme homolog, voltage-dependent T-type calcium channel subunit alpha-1G, aldo-keto reductase family 1 member B1, voltage-dependent calcium channel gamma-4 subunit, adhesion molecule Dscam2, tudor domain-containing protein 1-like, thrombospondin type-1 domain-containing protein 7A, guanylate cyclase 32E, dynein axonemal heavy chain 3, myosin-G heavy chain-like, DNA-directed RNA polymerase II, troponin C, sodium-coupled monocarboxylate transporter 1, cGMP-dependent protein kinase, ATP-binding cassette sub-family C member Sur-like, sterol O-acyltransferase 1, nitric oxide synthase-like protein, NOX, Bardet-Biedl syndrome 1 protein, ATP-dependent RNA helicase DHH1, progestin and adipoQ receptor, chitin deacetylase 4, aminoacylase-1-like, palmitoyltransferase, dnaJ homolog subfamily C member 10, proton-associated sugar transporter A, myrosinase 1 , acetylcholine receptor subunit alpha-like, fasciclin-1, sorbin and SH3 domain-containing protein 1 , aminopeptidase N (apn2), cyclic nucleotide-gated cation channel subunit A-like, glutamate receptor 1-like, fatty acyl reductase (FAR7), peroxidasin-like, potassium channel subfamily T member 2, trehalose transporter Tret1-like , endothelin-converting enzyme, neuralized-like protein 4, dynein heavy chain 6, sodium/potassium/calcium exchanger 4-like, sodium/potassium/calcium exchanger 3, mucin-5AC, GATOR complex protein NPRL2-like , ubiquitin-protein ligase MYCBP2
20 (GR11)	8,042	6	beta-alanine transporter, photoreceptor-specific nuclear receptor, trichohyalin, GILT-like protein 1, arginine N-methyltransferase 9-like, 5-aminolevulinate synthase, erythroid-specific

21 (GR12)	7,744	26	ATP-dependent RNA helicase DDX18-like, delta-1-pyrroline-5-carboxylate synthase, UDP-glycosyltransferase 42B5, tubulin-specific chaperone cofactor E-like protein, tubulin-specific chaperone cofactor E-like protein, ADAM 17-like protease, elongation of very long chain fatty acids protein 7-like, glucosamine 6-phosphate N-acetyltransferase, N-alpha-acetyltransferase 60, serine/threonine-protein kinase Tor, delta and Notch-like epidermal growth factor-related receptor, glucose-1-phosphatase-like, ras-responsive element-binding protein 1, tyrosine phosphatase domain-containing protein, enhancer protein ISL-1, serine protease easter-like, anoctamin-8-like, nuclear hormone receptor HR3, aldo-keto reductase family 1 member B1, transcription factor 3C polypeptide 6-like, aminoacylase-1-like, lipase 3-like, myelin transcription factor 1, acyl-CoA Delta-9 desaturase, tyrosine kinase receptor Cad96Ca, paramyosin-like
22 (SP)	5,711	34	signal peptidase complex subunit 3, peroxidase-like, tonB2-like, collagenase-like, brachyurin-like, chymotrypsin-1-like, pancreatic triacylglycerol lipase-like, alpha-glucosidase, omega-amidase NIT2, ADP-ribosylation factor-like protein 5A, lipase member H-like, trypsin, alkaline C-like, 5-formyltetrahydrofolate cyclo-ligase, venom carboxylesterase-6-like, caspase-1-B-like, REPAT45, sodium/hydrogen exchanger 10-like, fatty acid-binding protein, collagenase-like, 4-coumarate--CoA ligase-like 5, uridine phosphorylase 1-like, pancreatic triacylglycerol lipase-like, anon-37Cs-like, transferrin-like, small integral membrane protein 12-A, leukocyte surface antigen CD53-like, fatty acid-binding protein, mitochondrial dicarboxylate carrier, lipase 1-like, syntaxin-like, arrestin domain-containing protein 5-like, retinoid-inducible serine carboxypeptidase-like, aminopeptidase N5
23 (nHC5)	3,944	50	speckle-type POZ protein-like, organic cation transporter protein-like, putative cyclin-dependent serine/threonine-protein kinase, myosuppressin, mediator of RNA polymerase II transcription subunit 24, pseudouridine-5'-phosphatase, potassium channel subfamily K member 1, cysteine--tRNA ligase, adhesion G protein-coupled receptor A3, methyl-CpG-binding domain protein 3, SAGA-associated factor 11 homolog, sec1 family domain-containing protein 2-like, ATP-dependent RNA helicase DDX42, GTP-binding protein REM 1, BTB/POZ domain-containing protein 6-B, gamma-glutamyl hydrolase A-like, centrosomal protein, SET domain-containing protein SmydA-8, calphotin-like, gamma-tubulin complex component 4, alpha-tocopherol transfer protein-like, microprocessor complex subunit DGCR8, TBC1 domain family member 7, fumarylacetoacetate hydrolase domain-containing protein 2, proteasome subunit alpha type-5, lysozyme, YY1-associated factor 2, alpha-N-acetylgalactosaminidase, electron transfer flavoprotein beta subunit lysine methyltransferase-like, nuclear factor 1 B-type, hemicentin-2, pyruvate dehydrogenase (acetyl-transferring) kinase, cyclin-dependent kinase 6, guanine nucleotide-binding protein-like 1, monocarboxylate transporter 12-like, lava lamp-like, transcription factor Ouib-like, dual specificity protein phosphatase MPK-4, ankyrin repeat domain-containing protein 50-like, fatty acyl reductase (FAR1), scavenger receptor class B member 1, lin-28 homolog, succinyl-CoA:3-ketoacid coenzyme A transferase 1, venom serine carboxypeptidase, post-GPI attachment to proteins factor 3, fatty acyl-CoA reductase 1-like, T-cell activation inhibitor, mitochondrial, gamma-soluble NSF attachment protein, flightin

Table S3. Top three genes highly expressed in each hemocyte cluster of naïve *S. exigua* larvae

Cluster	Gene ID	Gene name	Annotation	p-value	Expression (FPKM)	% of cells expressing	% of cells in all cluster expressing
**0 (OE1)**	SPEXI_LOCUS5543	HP1	CAH0701009.1 unnamed protein product [*Spodoptera exigua*]	0.0	49.06	97.4	99.4
	SPEXI_LOCUS8825	40S RP S23	*Spodoptera frugiperda* 40S ribosomal protein S23 (LOC118262784), mRNA	1.02E-165	34.36	94.3	99.1
	SPEXI_LOCUS5542	HP2	CAH0701009.1 unnamed protein product [*Spodoptera exigua*]	0.0	33.85	87.7	86.5
**1 (nHC1)**	SPEXI_LOCUS11288	Procathepsin H	*Spodoptera frugiperda* pro-cathepsin H (LOC118267144), mRNA	0.0	577.73	99.9	82.3
	SPEXI_LOCUS6153	Cys proteinase 1-like	Spodoptera litura digestive cysteine proteinase 1-like (LOC111351029), mRNA	0.0	357.10	99.8	79.3
	SPEXI_LOCUS2768	HP3	Spodoptera litura uncharacterized LOC111355046 (LOC111355046), mRNA	0.0	318.43	99.9	95.2
**2 (GR1)**	SPEXI_LOCUS11054	HP5	-	0.0	114.27	99.9	75.5
	SPEXI_LOCUS820	SPARC1	*Spodoptera frugiperda* SPARC (LOC118269687), mRNA	0.0	74.09	73.8	94.8
	SPEXI_LOCUS3619	HP4	-	0.0	52.05	99.8	72.6
**3 (GR2)**	SPEXI_LOCUS8825	40S RP S23	*Spodoptera frugiperda* 40S ribosomal protein S23 (LOC118262784), mRNA	1.26E-191	50.86	92.6	94.8
	SPEXI_LOCUS8313	60S RP P1	*Spodoptera frugiperda* 60S acidic ribosomal protein P1 (LOC118262991), mRNA	1.30E-63	43.75	99.4	99.4
	SPEXI_LOCUS6878	Cercopin B	*Spodoptera exigua* cecropin B mRNA, complete cds	1.21E-294	34.07	99.5	98.8
**4 (PL1)**	SPEXI_LOCUS970	HP6	-	9.31E-286	77.31	56.0	42.9

	SPEXI_LOCUS5793	Paired mesoderm homeobox 2A-like	Spodoptera litura paired mesoderm homeobox protein 2A-like (LOC111358522), transcript variant X2, mRNA	8.31E-172	65.66	100	72.0
	SPEXI_LOCUS457	HP7	-	0.0	62.13	100	81.7
**5 (PL2)**	SPEXI_LOCUS10030	HP9	-	9.66E-160	146.84	99.74	49.4
	SPEXI_LOCUS10009	Slowmo	*Spodoptera frugiperda* protein slowmo (LOC118273107), mRNA	4.11E-74	142.24	98.95	56.0
	SPEXI_LOCUS10769	Mesencephalic astrocyte-derived neurotrophic factor homolog	*Spodoptera frugiperda* mesencephalic astrocyte-derived neurotrophic factor homolog (LOC118281782), mRNA	1.70E-52	92.24	98.95	59.6
**6 (nHC2)**	SPEXI_LOCUS35	Apoptosis-inducing factor	Spodoptera litura apoptosis-inducing factor 1, mitochondrial (LOC111354575), transcript variant X2, mRNA	0.0063	480.69	100	32.6
	SPEXI_LOCUS23	NEDD8	*Spodoptera frugiperda* NEDD8 ultimate buster 1-like (LOC118271503), mRNA	0.002	426.20	100	49.0
	SPEXI_LOCUS83	UBR1	*Spodoptera frugiperda* E3 ubiquitin-protein ligase UBR1 (LOC118271145), transcript variant X5, mRNA	0.0	291.40	93.9	36.8
**7 (GR3)**	SPEXI_LOCUS174	Hemicentin 1-like	Spodoptera litura hemicentin-1-like (LOC111362851), transcript variant X2, mRNA	0.0	85.43	99.7	29.6
	SPEXI_LOCUS8825	40S RP S23	*Spodoptera frugiperda* 40S ribosomal protein S23 (LOC118262784), mRNA	1.63E-170	36.12	100	94.8
	SPEXI_LOCUS6327	Sodium/calcium exchanger regulatory protein 1	*Spodoptera frugiperda* sodium/calcium exchanger regulatory protein 1 (LOC118274906), mRNA	2.85E-268	34.59	100.0	99.4

8 (GR4)	SPEXI_LOCUS8825	40S RP S23	*Spodoptera frugiperda* 40S ribosomal protein S23 (LOC118262784), mRNA	0.0	73.89	100.0	95.2
	SPEXI_LOCUS8313	60S RP P1	*Spodoptera frugiperda* 60S acidic ribosomal protein P1 (LOC118262991), mRNA	0.0	70.97	87.4	94.8
	SPEXI_LOCUS6327	Sodium/calcium exchanger regulatory protein 1	*Spodoptera frugiperda* sodium/calcium exchanger regulatory protein 1 (LOC118274906), mRNA	5.46E-110	66.95	100.0	99.4
9 (nHC3)	SPEXI_LOCUS11288	Procathepsin_H	*Spodoptera frugiperda* pro-cathepsin H (LOC118267144), mRNA	0.0	455.99	98.0	82.3
	SPEXI_LOCUS6153	Cys proteinase 1-like	Spodoptera litura digestive cysteine proteinase 1-like (LOC111351029), mRNA	0.0	343.90	96.7	79.3
	SPEXI_LOCUS2768	HP8	-	0.0	190.60	98.4	95.2
10 (GR5)	SPEXI_LOCUS8825	40S RP S23	*Spodoptera frugiperda* 40S ribosomal protein S23 (LOC118262784), mRNA	1.37E-65	43.58	100.0	99.4
	SPEXI_LOCUS8313	60S RP P1	*Spodoptera frugiperda* 60S acidic ribosomal protein P1 (LOC118262991), mRNA	3.09E-135	42.64	99.7	98.8
	SPEXI_LOCUS11358	RP L10	*Spodoptera exigua* ribosomal protein L10 (RpL10) mRNA, complete cds	9.06E-282	39.48	98.8	86.5
11 (OE2)	SPEXI_LOCUS11054	HP4	-	0.0	131.08	100.0	75.5
	SPEXI_LOCUS820	SPARC	*Spodoptera frugiperda* SPARC (LOC118269687), mRNA	0.0	85.94	100.0	72.6
	SPEXI_LOCUS10914	Hemocytin	*Spodoptera frugiperda* hemocytin (LOC118266761), mRNA	0.0	48.48	100.0	99.4
12 (GR6)	SPEXI_LOCUS3619	HP5	-	4.66E-169	66.45	100.0	94.8
	SPEXI_LOCUS8825	40S RP S23	*Spodoptera frugiperda* 40S	1.38E-66	52.77	96.9	80.1

			ribosomal protein S23 (LOC118262784), mRNA				
	SPEXI_LOCUS8313	60S RP P1	*Spodoptera frugiperda* 60S acidic ribosomal protein P1 (LOC118262991), mRNA	4.96E-166	46.61	98.4	45.9
**13 (OE3)**	SPEXI_LOCUS11054	HP4	-	0.0	125.36	75.5	94.4
	SPEXI_LOCUS820	SPARC	*Spodoptera frugiperda* SPARC (LOC118269687), mRNA	0.0	93.53	61.5	75.8
	SPEXI_LOCUS3619	HP5	-	0.0	83.75	100.0	75.5
**14 (GR7)**	SPEXI_LOCUS8825	40S RP S23	*Spodoptera frugiperda* 40S ribosomal protein S23 (LOC118262784), mRNA	5.03E-194	36.27	94.9	94.8
	SPEXI_LOCUS8313	60S RP	*Spodoptera frugiperda* 60S acidic ribosomal protein P1 (LOC118262991), mRNA	0.001	33.13	98.2	99.4
	SPEXI_LOCUS4004	GABA receptor	*Spodoptera frugiperda* gamma-aminobutyric acid receptor-associated protein (LOC118272711), mRNA	4.01E-183	32.96	98.6	98.8
**15 (GR8)**	SPEXI_LOCUS8825	40S RP S23	*Spodoptera frugiperda* 40S ribosomal protein S23 (LOC118262784), mRNA	4.19E-158	80.24	84.2	94.8
	SPEXI_LOCUS8313	60S RP	*Spodoptera frugiperda* 60S acidic ribosomal protein P1 (LOC118262991), mRNA	4.31E-171	78.78	100.0	99.4
	SPEXI_LOCUS6327	Sodium/calcium exchanger regulatory protein 1	*Spodoptera frugiperda* sodium/calcium exchanger regulatory protein 1 (LOC118274906), mRNA	0.0	59.96	100.0	98.8
**16 (GR9)**	SPEXI_LOCUS8825	40S RP S23	*Spodoptera frugiperda* 40S ribosomal protein S23 (LOC118262784), mRNA	1.01E-149	67.02	100.0	99.4
	SPEXI_LOCUS8313	60S RP P1	*Spodoptera*	2.88E-	57.20	100.0	98.8

			frugiperda 60S acidic ribosomal protein P1 (LOC118262991), mRNA	134			
	SPEXI_LOCUS7596	60S RP L23	Spodoptera litura 60S ribosomal protein L23 (LOC111350075), mRNA	1.54E-79	35.83	84.3	94.8
**17 (GR10)**	SPEXI_LOCUS8825	40S RP S23	*Spodoptera frugiperda* 40S ribosomal protein S23 (LOC118262784), mRNA	4.97E-29	51.29	94.8	45.2
	SPEXI_LOCUS8313	60S RP P1	*Spodoptera frugiperda* 60S acidic ribosomal protein P1 (LOC118262991), mRNA	1.65E-23	47.57	99.6	99.0
	SPEXI_LOCUS5256	EF1 alph-like	*Spodoptera frugiperda* elongation factor 1-alpha-like (LOC118272072), transcript variant X1, mRNA	2.47E-96	46.34	99.6	99.4
**18 (nHC4)**	SPEXI_LOCUS11288	Procathepsin H	*Spodoptera frugiperda* pro-cathepsin H (LOC118267144), mRNA	4.41E-83	465.79	100.0	82.3
	SPEXI_LOCUS6153	Cys proteinase 1-like	Spodoptera litura digestive cysteine proteinase 1-like (LOC111351029), mRNA	1.07E-74	332.44	100.0	95.2
	SPEXI_LOCUS2768	HP3	Spodoptera litura uncharacterized LOC111355046 (LOC111355046), mRNA	0.0	239.41	100.0	79.3
**19 (OE4)**	SPEXI_LOCUS5543	HP1	-	1.74E-108	51.18	95.7	99.4
	SPEXI_LOCUS5542	HP2	-	6.53E-99	33.22	92.3	93.7
	SPEXI_LOCUS8825	40S RP S23	*Spodoptera frugiperda* 40S ribosomal protein S23 (LOC118262784), mRNA	1.32E-158	27.79	81.8	95.2
**20 (GR11)**	SPEXI_LOCUS173	Hemicentin 2-like	Spodoptera litura hemicentin-2-like (LOC111354572), mRNA	0.0	212.23	79.5	94.8
	SPEXI_LOCUS5407	BLL4	*Spodoptera exigua* BLL4 (BLL4) mRNA, complete cds	0.0	59.77	98.3	28.1
	SPEXI_LOCUS11970	HSP70 cognate 4	*Spodoptera frugiperda* heat shock 70 kDa	2.31E-136	55.42	89.8	54.8

			protein cognate 4 (LOC118268613), mRNA				
21 (GR12)	SPEXI_LOCUS174	Hemicentin 1-like	Spodoptera litura hemicentin-1-like (LOC111362851), transcript variant X2, mRNA	3.52E-103	121.44	75.3	94.8
	SPEXI_LOCUS8825	40S RP S23	*Spodoptera frugiperda* 40S ribosomal protein S23 (LOC118262784), mRNA	4.68E-150	34.36	100.0	29.6
	SPEXI_LOCUS8313	60S RP P1	*Spodoptera frugiperda* 60S acidic ribosomal protein P1 (LOC118262991), mRNA	0.0	26.46	70.1	80.1
22 (SP)	SPEXI_LOCUS8313	60S RP P1	*Spodoptera frugiperda* 60S acidic ribosomal protein P1 (LOC118262991), mRNA	0.0	59.65	100.0	53.2
	SPEXI_LOCUS6327	Sodium/calcium exchanger regulatory protein 1	*Spodoptera frugiperda* sodium/calcium exchanger regulatory protein 1 (LOC118274906), mRNA	0.0	47.63	98.1	31.2
	SPEXI_LOCUS8825	40S RP S23	*Spodoptera frugiperda* 40S ribosomal protein S23 (LOC118262784), mRNA	1.34E-101	45.68	78.2	41.4
23 (nHC5)	SPEXI_LOCUS6903	Myosin regulatory light chain 2	Spodoptera litura myosin regulatory light chain 2 (LOC111356993), mRNA	7.96E-269	154.36	92.3	61.9
	SPEXI_LOCUS349	Troponin T	*Spodoptera frugiperda* troponin T, skeletal muscle (LOC118263234), transcript variant X7, mRNA	0.0	135.77	100.0	82.3
	SPEXI_LOCUS726	Troponin I	*Spodoptera frugiperda* troponin I (LOC118263900), transcript variant X12, mRNA	5.62E-65	109.98	100.0	32.3

Table S4. Top three genes highly expressed in each hemocyte cluster of immune-challenged *S. exigua* larvae

Cluster	Gene ID	Gene name	Annotation	p-value	Expression (FPKM)	% of cells in cluster expressing	% of cells in all cluster expressing
0 (OE1)	SPEXI_LOCUS8825	40S RP S23	*Spodoptera frugiperda* 40S ribosomal protein S23	4.69E-51	42.65	99.2	99.9
	SPEXI_LOCUS3864	RP L18	*Spodoptera frugiperda* ribosomal protein L18 mRNA, complete cds	2.75E-55	30.60	97.4	99.4
	SPEXI_LOCUS3619	HP7	-	2.64E-29	29.19	91.2	86.5
1 (nHC1)	SPEXI_LOCUS11288	Procathepsin H	*Spodoptera frugiperda* pro-cathepsin H (LOC118267144), mRNA	4.91E-26	653.15	99.9	82.3
	SPEXI_LOCUS6153	Cys proteinase 1-like	Spodoptera litura digestive cysteine proteinase 1-like (LOC111351029), mRNA	0.71	413.74	100	79.3
	SPEXI_LOCUS6327	Sodium/calcium exchanger regulatory protein 1	*Spodoptera frugiperda* sodium/calcium exchanger regulatory protein 1 (LOC118274906), mRNA	2.23E-25	259.91	100	95.2
2 (GR1)	SPEXI_LOCUS11054	HP	-.	6.68E-44	89.14	99.0	75.4
	SPEXI_LOCUS6878	Cercopin B	*Spodoptera exigua* cecropin B mRNA, complete cds	0.0	87.96	99.5	94.8
	SPEXI_LOCUS820	SPARC	*Spodoptera frugiperda* SPARC (LOC118269687), mRNA	5.23E-10	68.23	99.1	72.6
3 (GR2)	SPEXI_LOCUS6878	Cercopin B	*Spodoptera exigua* cecropin B mRNA, complete cds	6.90E-93	131.51	98.7	94.8
	SPEXI_LOCUS8825	40S RP S23	PREDICTED: *Spodoptera frugiperda* 40S ribosomal protein S23 (LOC118262784), mRNA	5.78E-06	49.12	96.3	99.3
	SPEXI_LOCUS8313	60S RP 1	*Spodoptera frugiperda* 60S acidic ribosomal protein P1 (LOC118262991), mRNA	0.009	45.34	96.3	98.7

**4 (PL1)**	SPEXI_LOCUS970	HP6	*Spodoptera exigua* genome assembly, chromosome: 11	0.0008	76.99	55.3	41.0
	SPEXI_LOCUS842	HP8	*Spodoptera exigua* genome assembly, chromosome: 11	0.14	60.40	39.1	52.01
	SPEXI_LOCUS579	Paired mesoderm homeobox 2A-like	Spodoptera litura paired mesoderm homeobox protein 2A-like (LOC111358522), transcript variant X2, mRNA	0.0005	58.79	48.5	37.0
**5 (PL2)**	SPEXI_LOCUS10009	Slowmo	*Spodoptera frugiperda* protein slowmo (LOC118273107), mRNA	0.63	165.83	59.56	49.3
	SPEXI_LOCUS10030	HP9	coverpa zea isolate GA-R chromosome 4	6.48E-06	147.24	61.0	25.3
	SPEXI_LOCUS10769	Mesencephalic astrocyte-derived neurotrophic factor homolog	*Spodoptera frugiperda* mesencephalic astrocyte-derived neurotrophic factor homolog (LOC118281782), mRNA	1.27E-43	70.77	57.0	59.6
**6 (nHC2)**	SPEXI_LOCUS3559	Apoptosis-inducing factor	Spodoptera litura apoptosis-inducing factor 1, mitochondrial (LOC111354575), transcript variant X2, mRNA	0.0002	568.86	100.0	32.5
	SPEXI_LOCUS23	NEDD8	*Spodoptera frugiperda* NEDD8 ultimate buster 1-like (LOC118271503), mRNA	0.015	404.56	71.4	38.9
	SPEXI_LOCUS83	UBR1	*Spodoptera frugiperda* E3 ubiquitin-protein ligase UBR1 (LOC118271145), transcript variant X5, mRNA	0.015	265.79	91.9	56.7
**7 (GR3)**	SPEXI_LOCUS174	Hemicentin 1-like	Spodoptera litura hemicentin-1-like (LOC111362851), transcript variant X2, mRNA	0.44	73.17	99.7	29.5
	SPEXI_LOCUS6878	Cecropin B	*Spodoptera exigua* cecropin B mRNA, complete cds	3.52E-15	49.12	98.8	94.8
	SPEXI_LOCUS8825	40S RP S23	*Spodoptera frugiperda* 40S ribosomal protein S23 (LOC118262784),	2.01E-12	29.36	100	99.3

			mRNA				
**8 (GR4)**	SPEXI_LOCUS6327	Sodium/calcium exchanger regulatory protein 1	*Spodoptera frugiperda* sodium/calcium exchanger regulatory protein 1 (LOC118274906), mRNA	0.18	78.39	100	95.2
	SPEXI_LOCUS6878	Cecropin B	*Spodoptera exigua* cecropin B mRNA, complete cds	2.14E-103	70.48	99.8	94.8
	SPEXI_LOCUS8825	40S RP S23	*Spodoptera frugiperda* 40S ribosomal protein S23 (LOC118262784), mRNA	2.19E-07	70.44	100	99.3
**9 (nHC3)**	SPEXI_LOCUS11288	Procathepsin H	*Spodoptera frugiperda* pro-cathepsin H (LOC118267144), mRNA	2.35E-127	517.53	97.2	82.3
	SPEXI_LOCUS6153	Cys proteinase 1-like	Spodoptera litura digestive cysteine proteinase 1-like (LOC111351029), mRNA	4.12E-140	349.99	98.75	79.3
	SPEXI_LOCUS6327	Sodium/calcium exchanger regulatory protein 1	*Spodoptera frugiperda* sodium/calcium exchanger regulatory protein 1 (LOC118274906), mRNA	3.77E-104	188.44	99.5	95.2
**10 (GR5)**	SPEXI_LOCUS8825	40S RP S23	*Spodoptera frugiperda* 40S ribosomal protein S23 (LOC118262784), mRNA	1.46E-75	47.14	100	99.3
	SPEXI_LOCUS8313	60S RP P1	*Spodoptera frugiperda* 60S acidic ribosomal protein P1 (LOC118262991), mRNA	2.76E-90	44.00	100	98.7
	SPEXI_LOCUS3619	HP5	*Spodoptera exigua* genome assembly, chromosome: 5	3.44E-102	41.94	98.2	86.5
**11 (OE2)**	SPEXI_LOCUS11054	HP2	-.	1.42E-70	79.75	100	75.4
	SPEXI_LOCUS820	SPARC2	*Spodoptera frugiperda* SPARC (LOC118269687), mRNA	1.25E-29	55.76	100	72.6
	SPEXI_LOCUS8825	40S RP S23	*Spodoptera frugiperda* 40S ribosomal protein S23	0.33	44.84	100	99.39

			(LOC118262784), mRNA				
**12 (GR6)**	SPEXI_LOCUS6878	Cecropin B	*Spodoptera exigua* cecropin B mRNA, complete cds	2.26E-33	211.63	100	94.8
	SPEXI_LOCUS6881	Cecropin A1	*Spodoptera exigua* cecropin A1 mRNA, complete cds	3.54E-34	54.86	100	80.1
	SPEXI_LOCUS6461	PGRP LB-like	Spodoptera litura peptidoglycan-recognition protein LB-like (LOC111360326), mRNA	1.09E-19	42.07	99.8	45.9
**13 (OE3)**	SPEXI_LOCUS6221	PPO1	*Spodoptera exigua* prophenoloxidase-1 mRNA, complete cds	0.0001	126.36	92.3	94.4
	SPEXI_LOCUS6527	PPO2	*Spodoptera exigua* prophenoloxidase mRNA, complete cds	0.035	115.94	71.2	75.8
	SPEXI_LOCUS11054	HP3	-.	2.13E-18	73.68	99.5	75.4
**14 (GR7)**	SPEXI_LOCUS6878	Cecropin B	*Spodoptera exigua* cecropin B mRNA, complete cds	2.10E-55	49.70	95.6	94.8
	SPEXI_LOCUS8825	40S RP S23	*Spodoptera frugiperda* 40S ribosomal protein S23 (LOC118262784), mRNA	0.0002	32.40	98.3	99.3
	SPEXI_LOCUS8313	60S RP	*Spodoptera frugiperda* 60S acidic ribosomal protein P1 (LOC118262991), mRNA	0.0003	28.61	98.0	98.7
**15 (GR8)**	SPEXI_LOCUS6878	Cecropin B	*Spodoptera exigua* cecropin B mRNA, complete cds	1.77E-24	106.94	100	94.8
	SPEXI_LOCUS8825	40S RP S23	*Spodoptera frugiperda* 40S ribosomal protein S23 (LOC118262784), mRNA	4.68E-05	71.23	100	99.3
	SPEXI_LOCUS8313	60S RP P1	*Spodoptera frugiperda* 60S acidic ribosomal protein P1 (LOC118262991), mRNA	0.018	70.16	98.9	98.7
**16 (GR9)**	SPEXI_LOCUS8825	40S RP S23	*Spodoptera frugiperda* 40S ribosomal protein S23	7.60E-124	67.53	100	99.3

			(LOC118262784), mRNA				
	SPEXI_LOCUS8313	60S RP P1	*Spodoptera frugiperda* 60S acidic ribosomal protein P1 (LOC118262991), mRNA	0.0009	58.82	100	98.7
	SPEXI_LOCUS6878	Cecropin B	*Spodoptera exigua* cecropin B mRNA, complete cds	1.21E-62	41.48	100	94.8
**17 (GR10)**	SPEXI_LOCUS6878	Cecropin B	*Spodoptera exigua* cecropin B mRNA, complete cds	9.29E-41	60.50	99.1	94.8
	SPEXI_LOCUS5256	EF1 alpha-like	*Spodoptera frugiperda* elongation factor 1-alpha-like (LOC118272072), transcript variant X1, mRNA	5.66E-27	46.27	100	98.9
	SPEXI_LOCUS8825	40S RP S23	*Spodoptera frugiperda* 40S ribosomal protein S23 (LOC118262784), mRNA	6.33E-06	42.36	100	99.3
**18 (nHC4)**	SPEXI_LOCUS11288	Procathepsin H	*Spodoptera frugiperda* pro-cathepsin H (LOC118267144), mRNA	3.55E-67	216.00	98.9	82.3
	SPEXI_LOCUS6327	Sodium/calcium exchanger regulatory protein 1	*Spodoptera frugiperda* sodium/calcium exchanger regulatory protein 1 (LOC118274906), mRNA	3.29E-98	207.73	100	95.2
	SPEXI_LOCUS6153	Cys proteinase 1-like	Spodoptera litura digestive cysteine proteinase 1-like (LOC111351029), mRNA	1.21E-62	128.55	98.1	79.3
**19 (OE4)**	SPEXI_LOCUS8825	40S RP S23	*Spodoptera frugiperda* 40S ribosomal protein S23 (LOC118262784), mRNA	1.19E-07	54.55	98.4	99.3
	SPEXI_LOCUS8264	60S RP	*Spodoptera frugiperda* 60S acidic ribosomal protein P0 (LOC118262435), mRNA	1.14E-33	46.90	86.3	93.6
	SPEXI_LOCUS6327	Sodium/calcium exchanger regulatory protein 1	*Spodoptera frugiperda* sodium/calcium exchanger regulatory protein 1	2.89E-06	39.02	95.4	95.2

			(LOC118274906), mRNA				
**20 (GR11)**	SPEXI_LOCUS6878	Cecropin B	*Spodoptera exigua* cecropin B mRNA, complete cds	7.20E-44	235.88	98.1	94.8
	SPEXI_LOCUS173	Hemicentin 2-like	Spodoptera litura hemicentin-2-like (LOC111354572), mRNA	1.27E-08	95.89	97.4	58.0
	SPEXI_LOCUS9978	Spodoptericin	*Spodoptera exigua* spodoptericin mRNA, complete cds	1.85E-43	60.01	93.7	54.8
**21 (GR12)**	SPEXI_LOCUS6878	Cecropin B	*Spodoptera exigua* cecropin B mRNA, complete cds	2.99E-131	163.58	100	94.8
	SPEXI_LOCUS174	Hemicentin 1-like	Spodoptera litura hemicentin-1-like (LOC111362851), transcript variant X2, mRNA	1.14E-33	81.67	100	49.5
	SPEXI_LOCUS6881	cecropin A1	*Spodoptera exigua* cecropin A1 mRNA, complete cds	4.49E-06	39.64	94.5	80.1
**22 (SP)**	SPEXI_LOCUS5733	Calphotin-like	*Spodoptera frugiperda* calphotin-like (LOC118266478), mRNA	2.59E-11	184.60	100	63.2
	SPEXI_LOCUS5215	Trypsin alkB	*Spodoptera frugiperda* trypsin, alkaline B (LOC118274829), mRNA	1.51E-13	60.14	83.33	51.1
	SPEXI_LOCUS11234	HP12	*Spodoptera exigua* genome assembly, chromosome: 7	1.85E-43	58.95	100	41.3
**23 (nHC5)**	SPEXI_LOCUS4419	HP13	*Spodoptera exigua* genome assembly, chromosome: 19	2.99E-131	120.88	92.3	71.8
	SPEXI_LOCUS11288	Procathepsin H	*Spodoptera frugiperda* pro-cathepsin H (LOC118267144), mRNA	0.4480	100.09	84.6	82.3
	SPEXI_LOCUS6903	Myosin regulatory light chain 2	Spodoptera litura myosin regulatory light chain 2 (LOC111356993), mRNA	3.83E-127	91.21	100	62.3

(A)

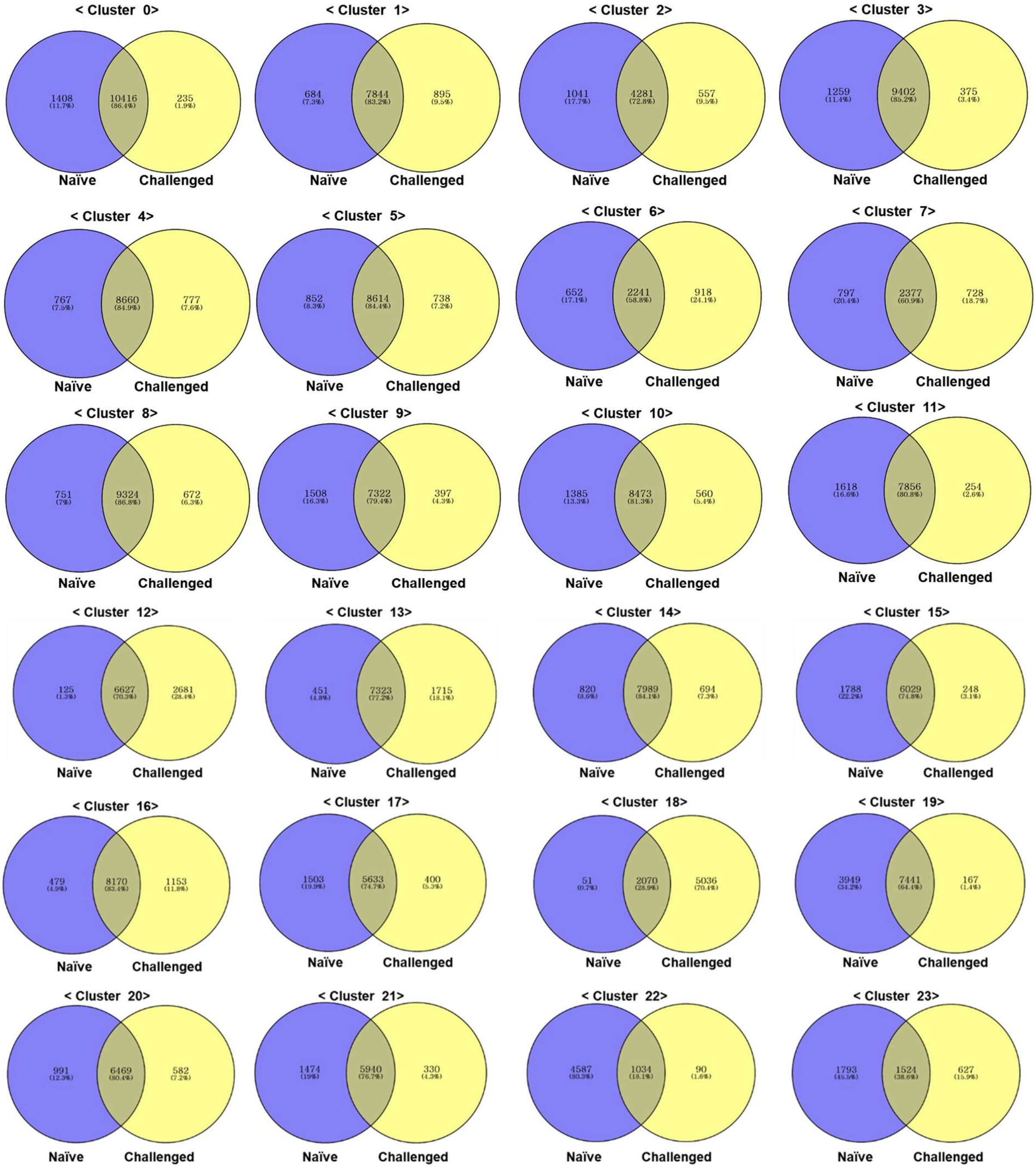

< Cluster 0>
1408 (11.7%)
10416 (86.4%)
235 (1.9%)
Naïve
Challenged
< Cluster 1>
684 (7.3%)
7844 (83.2%)
895 (9.5%)
Naïve
Challenged
< Cluster 2>
1041 (17.7%)
4281 (72.8%)
557 (9.5%)
Naïve
Challenged
< Cluster 3>
1259 (11.4%)
9402 (85.2%)
375 (3.4%)
Naïve
Challenged
< Cluster 4>
767 (7.5%)
8660 (84.9%)
777 (7.6%)
Naïve
Challenged
< Cluster 5>
852 (8.3%)
8614 (84.4%)
738 (7.2%)
Naïve
Challenged
< Cluster 6>
652 (17.1%)
2241 (58.8%)
918 (24.1%)
Naïve
Challenged
< Cluster 7>
797 (20.4%)
2377 (60.9%)
728 (18.7%)
Naïve
Challenged
< Cluster 8>
751 (7%)
9324 (86.8%)
672 (6.3%)
Naïve
Challenged
< Cluster 9>
1508 (16.3%)
7322 (79.4%)
397 (4.3%)
Naïve
Challenged
< Cluster 10>
1385 (13.3%)
8473 (81.3%)
560 (5.4%)
Naïve
Challenged
< Cluster 11>
1618 (16.6%)
7856 (80.8%)
254 (2.6%)
Naïve
Challenged
< Cluster 12>
125 (1.3%)
6627 (70.3%)
2681 (28.4%)
Naïve
Challenged
< Cluster 13>
451 (4.8%)
7323 (77.2%)
1715 (18.1%)
Naïve
Challenged
< Cluster 14>
820 (8.6%)
7989 (84.1%)
694 (7.3%)
Naïve
Challenged
< Cluster 15>
1788 (22.2%)
6029 (74.8%)
248 (3.1%)
Naïve
Challenged
< Cluster 16>
479 (4.9%)
8170 (83.4%)
1153 (11.8%)
Naïve
Challenged
< Cluster 17>
1503 (19.9%)
5633 (74.7%)
400 (5.3%)
Naïve
Challenged
< Cluster 18>
51 (0.7%)
2070 (28.9%)
5036 (70.4%)
Naïve
Challenged
< Cluster 19>
3949 (34.2%)
7441 (64.4%)
167 (1.4%)
Naïve
Challenged
< Cluster 20>
991 (12.3%)
6469 (80.4%)
582 (7.2%)
Naïve
Challenged
< Cluster 21>
1474 (19%)
5940 (76.7%)
330 (4.3%)
Naïve
Challenged
< Cluster 22>
4587 (80.3%)
1034 (18.1%)
90 (1.6%)
Naïve
Challenged
< Cluster 23>
1793 (45.5%)
1524 (38.6%)
627 (15.9%)
Naïve
Challenged

(B)

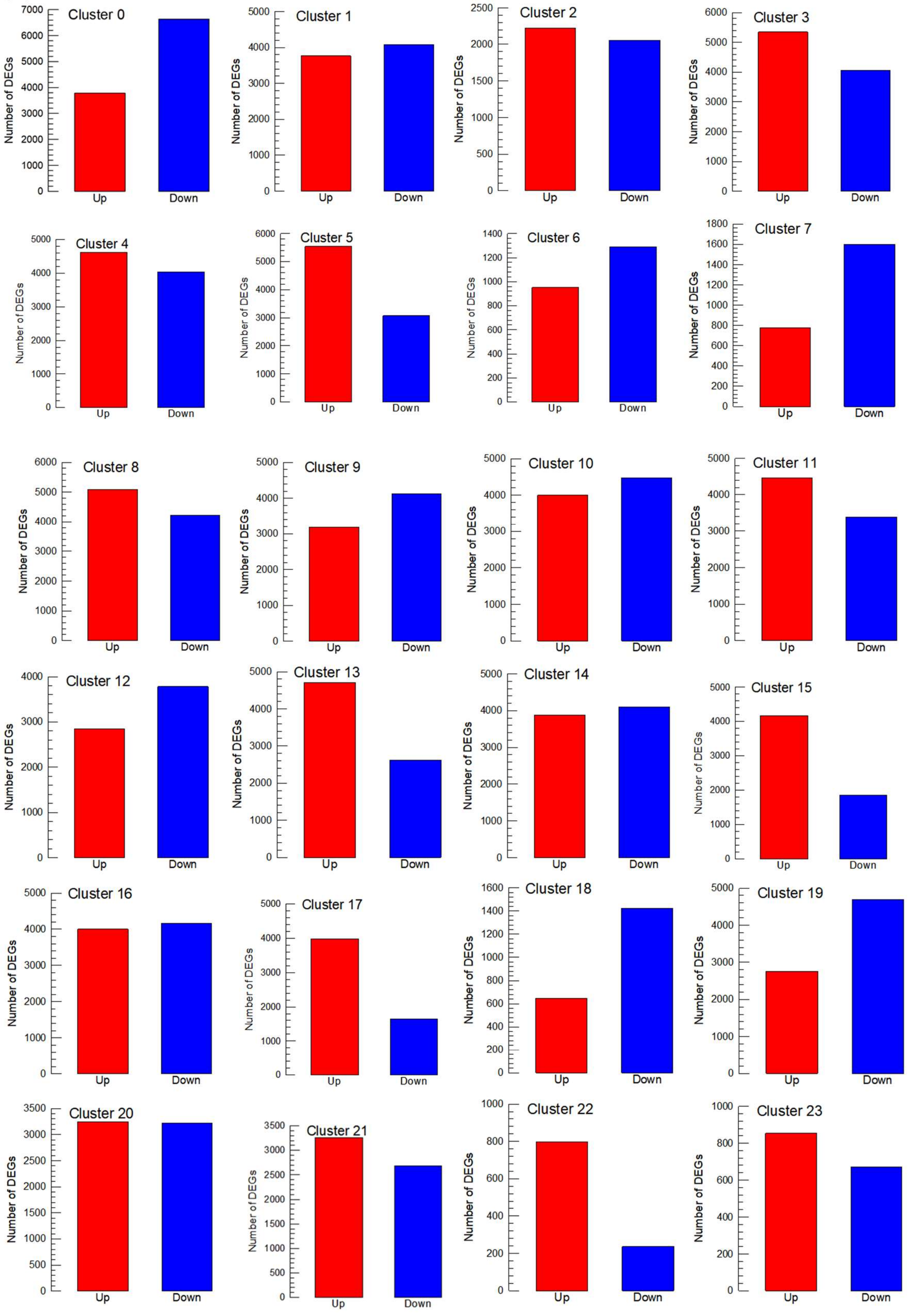
Cluster 0
Cluster 1
Cluster 2
Cluster 3
Cluster 4
Cluster 5
Cluster 6
Cluster 7
Cluster 8
Cluster 9
Cluster 10
Cluster 11
Cluster 12
Cluster 13
Cluster 14
Cluster 15
Cluster 16
Cluster 17
Cluster 18
Cluster 19
Cluster 20
Cluster 21
Cluster 22
Cluster 23
Number of DEGs
Up
Down

Fig. S1

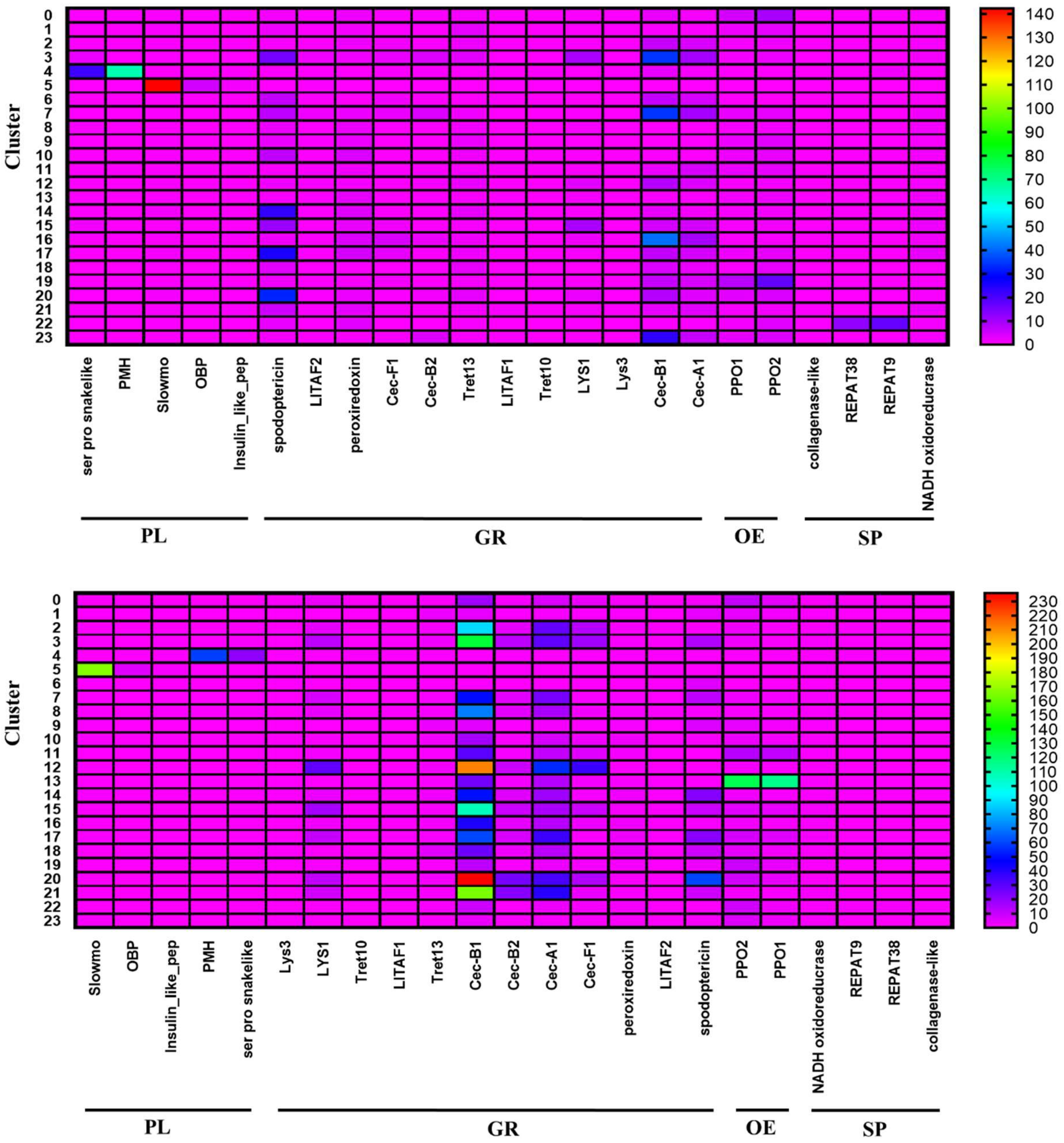
Cluster
ser pro snakelike
PMH
Slowmo
OBP
Insulin_like_pep
spodoptericin
LITAF2
peroxiredoxin
Cec-F1
Cec-B2
Tret13
LITAF1
Tret10
LYS1
Lys3
Cec-B1
Cec-A1
PPO1
PPO2
collagenase-like
REPAT38
REPAT9
NADH oxidoreducrase
PL
GR
OE
SP
Cluster
Slowmo
OBP
Insulin_like_pep
PMH
ser pro snakelike
Lys3
LYS1
Tret10
LITAF1
Tret13
Cec-B1
Cec-B2
Cec-A1
Cec-F1
peroxiredoxin
LITAF2
spodoptericin
PPO2
PPO1
NADH oxidoreducrase
REPAT9
REPAT38
collagenase-like
PL
GR
OE
SP

Fig. S2

(A) Rep 1:

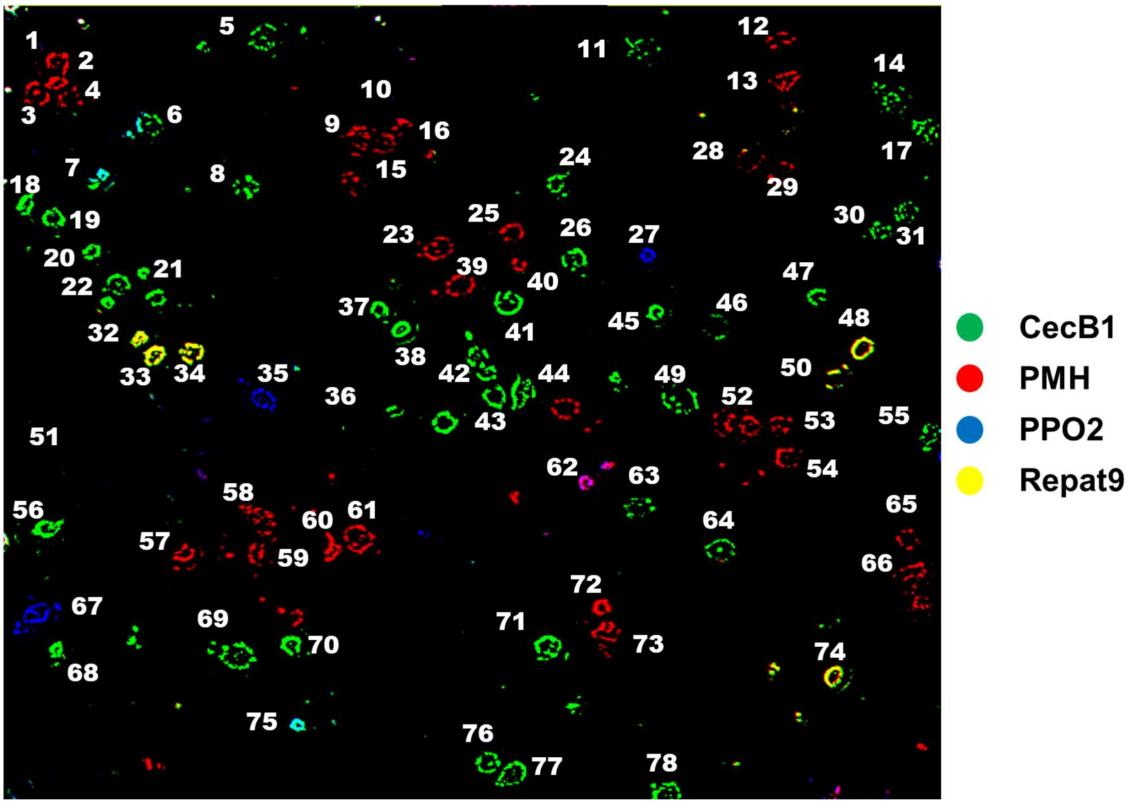

No of cells	Probe	No of cells	Probe	No of cells	Probe	No of cells	Probe
1	No signal	21	GR	41	GR	61	PL
2	PL	22	GR	42	GR	62	PL+SP
3	PL	23	PL	43	GR	63	GR
4	PL	24	GR	44	GR	64	GR
5	GR	25	PL	45	GR	65	PL
6	GR	26	GR	46	GR	66	PL
7	GR	27	SP	47	GR	67	SP
8	GR	28	PL	48	OE	68	GR
9	PL	29	PL	49	GR	69	GR
10	No signal	30	GR	50	OE	70	GR
11	GR	31	GR	51	No signal	71	GR
12	PL	32	OE	52	PL	72	PL
13	PL	33	OE	53	PL	73	PL
14	GR	34	OE	54	PL	74	OE
15	PL	35	SP	55	GR	75	GR+OE
16	PL	36	No signal	56	GR	76	GR
17	GR	37	GR	57	PL	77	GR
18	GR	38	GR	58	PL	78	GR
19	GR	39	PL	59	PL		
20	GR	40	PL	60	PL		

Rep 2:

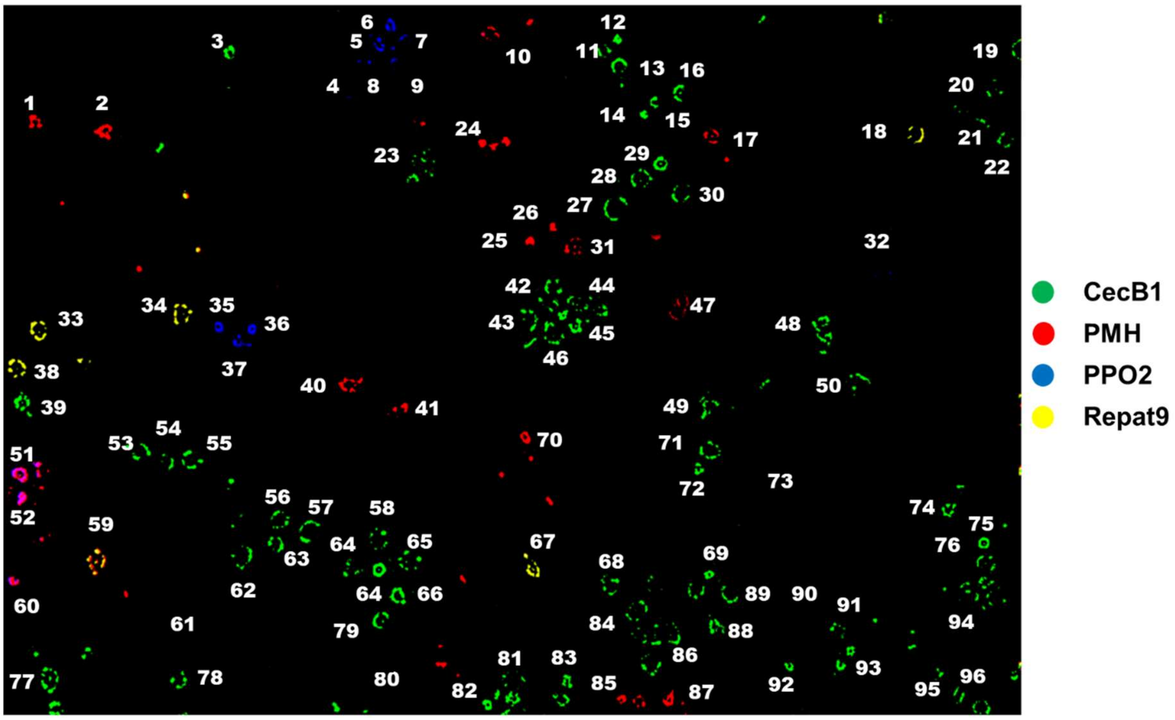

No of cells	Probe	No of cells	Probe	No of cells	Probe	No of cells	Probe
1	PL	25	PL	49	GR	73	No signal
2	PL	26	PL	50	GR	74	GR
3	GR	27	GR	51	PL+SP	75	GR
4	No signal	28	GR	52	PL+SP	76	GR
5	SP	29	GR	53	GR	77	GR
6	SP	30	GR	54	GR	78	GR
7	SP	31	PL	55	GR	79	GR
8	No signal	32	No signal	56	GR	80	No signal
9	No signal	33	OE	57	GR	81	GR
10	PL	34	OE	58	GR	82	PL
11	GR	35	SP	59	PL+OE	83	GR
12	GR	36	SP	60	PL+SP	84	GR
13	GR	37	SP	61	No signal	85	PL
14	GR	38	OE	62	GR	86	GR
15	GR	39	GR	63	GR	87	PL
16	GR	40	PL	64	GR	88	GR
17	PL	41	PL	65	GR	89	GR
18	SP	42	GR	66	GR	90	No signal
19	GR	43	GR	67	OE	91	GR
20	GR	44	GR	68	GR	92	GR
21	GR	45	GR	69	GR	93	GR
22	GR	46	GR	70	PL	94	GR
23	GR	47	PL	71	GR	95	GR
24	PL	48	GR	72	GR	96	GR

Rep 3:

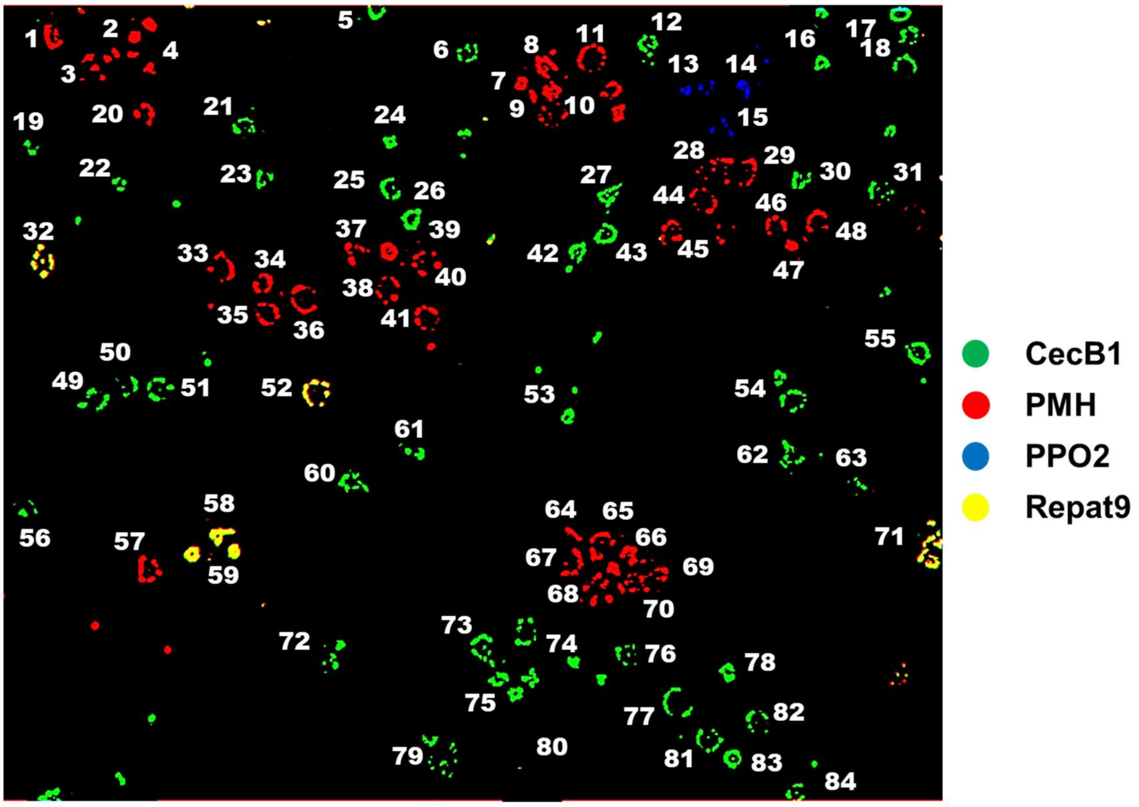

No of cells	Probe	No of cells	Probe	No of cells	Probe	No of cells	Probe
1	PL	22	GR	43	GR	64	PL
2	PL	23	GR	44	PL	65	PL
3	PL	24	GR	45	PL	66	PL
4	PL	25	GR	46	PL	67	PL
5	GR	26	GR	47	PL	68	PL
6	GR	27	GR	48	PL	69	PL
7	PL	28	PL	49	GR	70	PL
8	PL	29	PL	50	GR	71	OE
9	PL	30	GR	51	GR	72	GR
10	PL	31	GR	52	OE	73	GR
11	PL	32	OE	53	GR	74	GR
12	GR	33	PL	54	GR	75	GR
13	SP	34	PL	55	GR	76	GR
14	SP	35	PL	56	GR	77	GR
15	SP	36	PL	57	PL	78	GR
16	GR	37	PL	58	OE	79	GR
17	GR	38	PL	59	OE	80	No signal
18	GR	39	PL	60	GR	81	GR
19	PL	40	PL	61	GR	82	GR
20	GR	41	PL	62	GR	83	GR
21	GR	42	GR	63	GR	84	GR

(B)

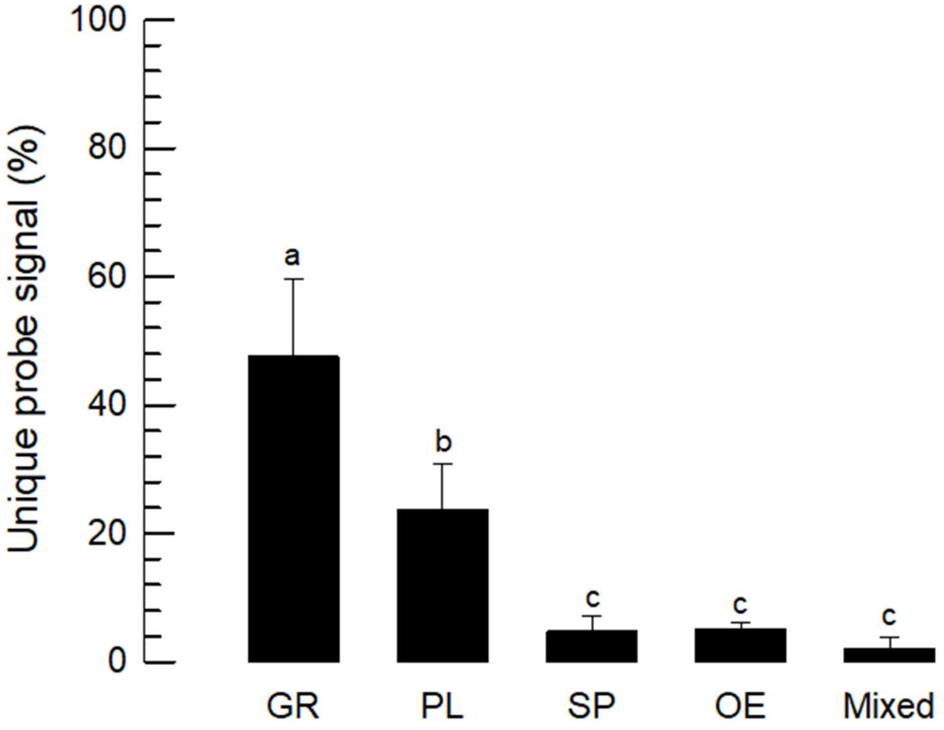

Fig. S3
